# Pseudomonas syringae Type III Secretion Protein HrpP Manipulates Plant Immunity To Promote Infection

**DOI:** 10.1128/spectrum.05148-22

**Published:** 2023-04-17

**Authors:** Ya Jin, Wei Zhang, Shen Cong, Qi-Guo Zhuang, Yi-Lin Gu, Yi-Nan Ma, Melanie J. Filiatrault, Jun-Zhou Li, Hai-Lei Wei

**Affiliations:** a Key Laboratory of Microbial Resources Collection and Preservation, Ministry of Agriculture and Rural Affairs, Institute of Agricultural Resources and Regional Planning, Chinese Academy of Agricultural Sciences, Beijing, China; b Plant Pathology and Plant-Microbe Biology Section, School of Integrative Plant Science, Cornell University, Ithaca, New York, USA; c Emerging Pests and Pathogens Research Unit, Agricultural Research Service, United States Department of Agriculture, Robert W. Holley Center for Agriculture and Health, Ithaca, New York, USA; d China-New Zealand Belt and Road Joint Laboratory on Kiwifruit, Kiwifruit Breeding and Utilization Key Laboratory of Sichuan Province, Sichuan Provincial Academy of Natural Resource Sciences, Chengdu, China; USDA-San Joaquin Valley Agricultural Sciences Center

**Keywords:** *Pseudomonas syringae*, type III secretion system, HrpP, pattern-triggered immunity, cell death, MKK2, *hrp* genes

## Abstract

The bacterial plant pathogen Pseudomonas syringae deploys a type III secretion system (T3SS) to deliver effector proteins into plant cells to facilitate infection, for which many effectors have been characterized for their interactions. However, few T3SS Hrp (hypersensitive response and pathogenicity) proteins from the T3SS secretion apparatus have been studied for their direct interactions with plants. Here, we show that the P. syringae pv. *tomato* DC3000 T3SS protein HrpP induces host cell death, suppresses pattern-triggered immunity (PTI), and restores the effector translocation ability of the *hrpP* mutant. The *hrpP*-transgenic *Arabidopsis* lines exhibited decreased PTI responses to flg22 and elf18 and enhanced disease susceptibility to P. syringae pv. *tomato* DC3000. Transcriptome analysis reveals that HrpP sensing activates salicylic acid (SA) signaling while suppressing jasmonic acid (JA) signaling, which correlates with increased SA accumulation and decreased JA biosynthesis. Both yeast two-hybrid and bimolecular fluorescence complementation assays show that HrpP interacts with mitogen-activated protein kinase kinase 2 (MKK2) on the plant membrane and in the nucleus. The HrpP truncation HrpP_1–119_, rather than HrpP_1–101_, retains the ability to interact with MKK2 and suppress PTI in plants. In contrast, HrpP_1–101_ continues to cause cell death and electrolyte leakage. MKK2 silencing compromises SA signaling but has no effect on cell death caused by HrpP. Overall, our work highlights that the P. syringae T3SS protein HrpP facilitates effector translocation and manipulates plant immunity to facilitate bacterial infection.

**IMPORTANCE** The T3SS is required for the virulence of many Gram-negative bacterial pathogens of plants and animals. This study focuses on the sensing and function of the T3SS protein HrpP during plant interactions. Our findings show that HrpP and its N-terminal truncation HrpP_1–119_ can interact with MKK2, promote effector translocation, and manipulate plant immunity to facilitate bacterial infection, highlighting the P. syringae T3SS component involved in the fine-tuning of plant immunity.

## INTRODUCTION

Plants have developed a complex and multilayered immune system to detect and repel pathogenic microbe invasion. Upon pathogen infection, cell surface-localized pattern recognition receptors (PRRs) perceive various pathogen-associated molecular patterns (PAMPs) and activate pattern-triggered immunity (PTI) (1). PTI activation induces basal defense responses such as a burst of reactive oxygen species (ROS), callose deposition, the activation of mitogen-activated protein kinase (MAPK) cascades, and the expression of defense-related genes (2–4). To circumvent PTI, plant pathogens deliver effectors into plant cells that interfere with PTI signaling and compromise PTI to enable pathogen proliferation (5, 6). In turn, plants have evolved nucleotide-binding leucine-rich-repeat (NLR) proteins that recognize effector proteins directly or indirectly and activate a second layer of innate immunity known as effector-triggered immunity (ETI), which usually results in hypersensitive response (HR)-like programmed cell death (7). Well-adapted pathogens secrete a battery of effectors that suppress host immunity and establish fitness, allowing host cell infection and resulting in effector-triggered susceptibility (ETS) (1). For Pseudomonas syringae and other plant bacterial pathogens, the type III secretion system (T3SS) behaves as a sophisticated macromolecular nanosyringe that secretes and delivers effector proteins to subvert innate immunity and remodel host cells for the benefit of the pathogen (5, 8, 9).

The T3SS in P. syringae is encoded by the hypersensitive response and pathogenicity/conserved (*hrp*-*hrc*) cluster, which has ca. 30 kb including approximately 20 genes (10). Structural studies of the T3SS injectisome revealed that this conserved nanomachine is composed of the following modules (11): the first is a basal body with two concentric protein rings that anchors the system to the bacterial membranes, the second is a needle/pilus filament that protrudes toward the outside of the bacterial surface and serves as a passageway for effector delivery, and the third is a translocation complex that allows the entry of virulence effectors into the plant cytoplasm. Previous studies showed that the P. syringae T3SS secreted some accessory or helper proteins to make up the extracellular portion of the type III apparatus and facilitate effector translocation (12–15). For example, the HrpA1 protein was shown to be the main component of the Hrp pilus (16, 17). Harpins (HrpZ1, HrpW, HopAK1, and HopP1) and HrpK1 apparently form the translocon that assists effectors in crossing the plant plasma membrane (PM) (18–21). Harpins are glycine-rich, cysteine-lacking proteins that were originally identified as HR inducers in nonhost plants when applied externally as purified proteins (20, 22–24). HrpZ1 proteins from various P. syringae pathovars bind to the outermost layers of the plant cell wall and associate with phosphatidic acid (21). A recent report also showed that the HrpZ1 protein interacts with cytoplastic host autophagy (ATG) proteins and enhances autophagy *in vivo* (25). The ectopic expression of *hrpZ1* in transgenic *Arabidopsis* plants makes the plants more susceptible to the *hrcC* mutant of P. syringae pv. *tomato* DC3000 (25). These findings highlight the potency of extracellular T3SS components in host manipulation.

Substantial work with animal pathogens has revealed that the assembly of the extracellular T3SS modules is governed by the length control protein YscP and other associated regulators (26). Mutation of *hpaC* and *hpaP*, the *yscP* homologs from the plant pathogens Xanthomonas campestris pv*. vesicatoria* and Ralstonia solanacearum, respectively, disrupt the secretion of effector proteins and T3SS substrates (27–30). Moreover, HpaC and HpaP are not secreted or translocated into plant cells (29). Similar to other T3SS-dependent pathogens, P. syringae pv. *tomato* DC3000 also harbors a YscP homolog, HrpP, which has a conserved type III secretion substrate specificity switch (T3S4) domain at its C terminus (31). The *hrpP* mutant fails to cause disease in tomato plants or elicit HR in tobacco plants; however, HrpP can be delivered into plant cells, and mutation of the *hrpP* gene reduces HrpA1 secretion and eliminates the secretion of other T3SS substrates (31). It has been hypothesized that YscP homologs play a key role in the early stages of type III secretion (30), which led us to consider whether HrpP could be perceived by host cells and how HrpP participates in host manipulation. Here, we show that purified HrpP proteins elicit plant cell death and suppress PTI reactions. We use transcriptome analysis to show that HrpP is involved in the regulation of immunity-associated protein kinase, transcription factor (TF), and hormone signaling. Furthermore, we show that *hrpP*-transgenic *Arabidopsis* enhances plant susceptibility to P. syringae pv. *tomato* DC3000. Finally, we demonstrate that HrpP can interact with plant MAPK kinase 2 (MKK2), relying on its N terminus to suppress plant immunity and promote infection.

## RESULTS

### HrpP-induced plant cell death and PTI inhibition.

**FIG 1:**
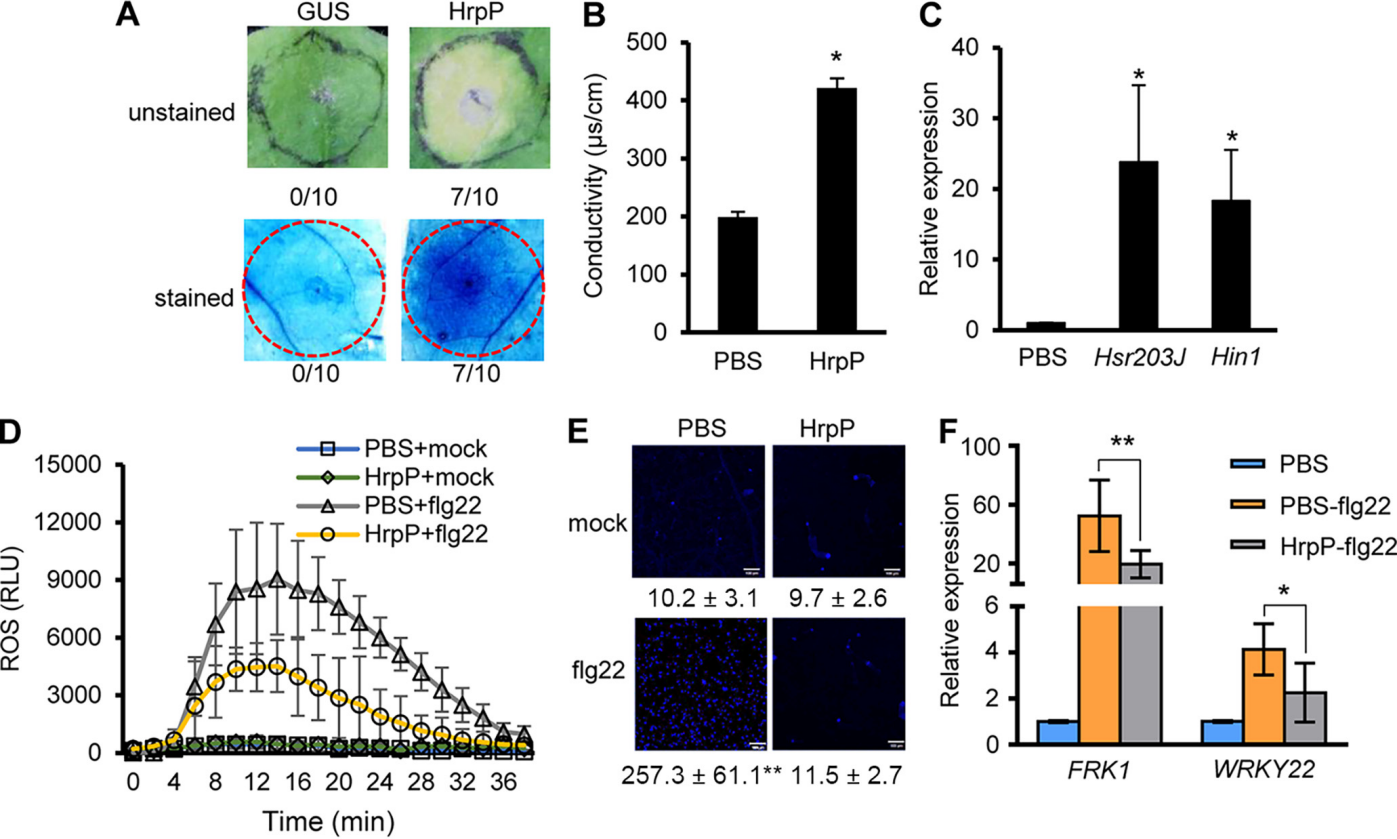
Purified HrpP protein elicits plant cell death and inhibits PTI. (A) Purified HrpP protein elicits cell death in N. benthamiana. Plant leaves were infiltrated with 20 μM HrpP or GUS protein. Cell death was assessed by tissue collapse at 2 dpi (top) and trypan blue staining at 1 dpi (bottom). The fraction below each image indicates the number of times that the HR was present relative to the number of test inoculations. (B) Conductivity was assayed 1 dpi. (C) Relative expression levels of cell death marker genes in N. benthamiana. Plant leaves were infiltrated with PBS or 1 μM HrpP protein, collected for total RNA isolation, and subsequently subjected to quantitative real-time PCR (qRT-PCR) assays at 6 hpi. NbEF-1α was used as the reference gene, and data were normalized to the values for the PBS control. (D) Purified HrpP protein inhibits the flg22-induced ROS burst in N. benthamiana. Plant leaves were infiltrated with PBS or 1 μM HrpP protein and collected at 6 hpi, and ROS accumulation was determined after treatment with 1 μM flg22. RLU, relative light units. (E) Purified HrpP protein inhibits flg22-induced callose deposition in N. benthamiana. Plant leaves were infiltrated with PBS or 1 μM HrpP protein, challenged with 1 μM flg22 at 6 hpi, and collected for callose examination after 15 h. Representative images from six biological replicates are presented. The numbers below each microscopy photograph indicate the average callose deposition and the standard error of the mean. (F) Relative expression of PTI marker genes in N. benthamiana. Plant leaves were infiltrated with PBS or 1 μM HrpP protein and challenged 3 h later by the inoculation of 1 μM flg22. Leaf disks were collected for qRT-PCR assays at 6 hpi. All of the experiments were repeated three times, with similar results. The data shown are the means ± standard deviations. * and ** indicate statistically significant differences (by a *t* test) at *P* values of <0.05 and <0.01, respectively.

Because the *hrpP* gene mutation affects effector protein translocation, the output in plants generated by the deficient *hrpP* mutant cannot be directly attributed to the absence of HrpP activity within the plant cell. To investigate the function of the HrpP protein, we produced HrpP proteins from Escherichia coli cells carrying an appropriate derivative of pET-30a, namely, a T7 expression vector with an N-terminal His_6_ tag fusion (see Fig. S1A in the supplemental material), and the purified HrpP protein was infiltrated into Nicotiana benthamiana for cell death elicitation assays. Remarkably, HrpP proteins elicited cell death-like tissue collapse in N. benthamiana at approximately 20 μM, while the β-glucuronidase (GUS) protein control elicited no visible response, which was also confirmed by trypan blue staining (Fig. 1A). Inoculation with HrpP proteins led to a significant increase in electrolyte leakage at 1 day postinoculation (dpi) (Fig. 1B) and the upregulation of two cell death marker genes, *Hsr203J* and *Hin1*, at 6 h postinoculation (hpi), compared with the mock (Fig. 1C). To assess the potential of HrpP to suppress PTI reactions, we conducted ROS and callose assays with 1 μM HrpP. Notably, HrpP suppressed the flg22-triggered ROS burst (Fig. 1D) and callose deposition (Fig. 1E) in N. benthamiana. Further tests showed that HrpP repressed the transcript accumulation of two PTI marker genes, *FRK1* and *WRKY22* (Fig. 1F). To examine whether HrpP might constrain PTI reactions in other plants, we repeated the ROS assay for *Arabidopsis* and Solanum lycopersicum. The results demonstrated that HrpP significantly inhibited the flg22-induced ROS burst in both plants (Fig. S1B and C). Collectively, these findings suggested that HrpP proteins can be sensed in the apoplast and can lead to the suppression of host innate immunity.

### HrpP sensing is essential for effector translocation and bacterial pathogenicity.

**FIG 2:**
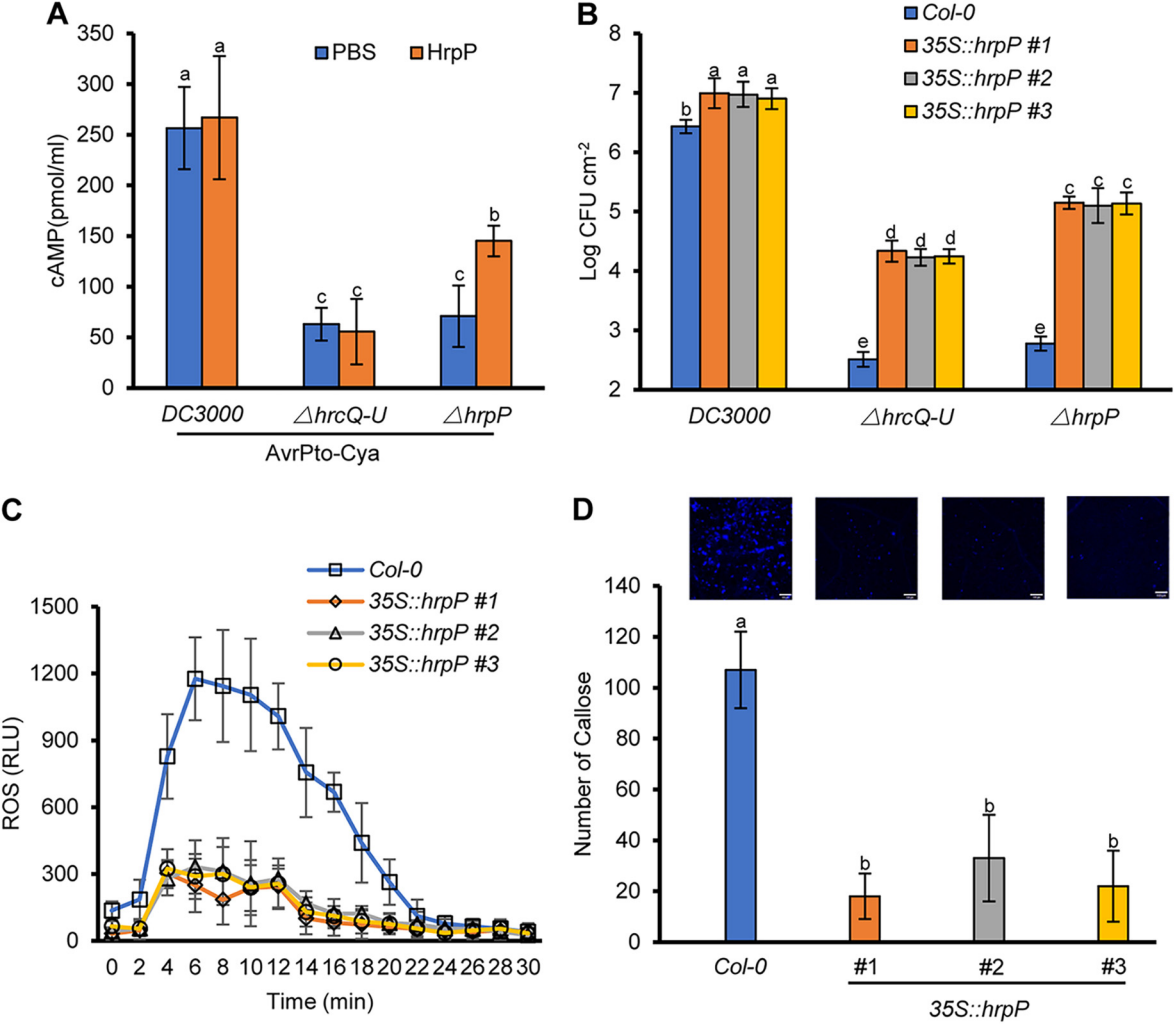
HrpP promotes effector translocation and bacterial infection. (A) Exogenous HrpP restored the effector translocation of the P. syringae pv. *tomato* DC3000 *hrpP* mutant. N. benthamiana leaves were infiltrated with PBS or 1 μM HrpP protein and challenged 6 h later by the inoculation of DC3000, DC3000 Δ*hrpP*, and DC3000 Δ*hrcQ–U* expressing AvrPto-Cya at 1 × 10^7^ CFU/mL. Leaf disks were collected for AvrPto-Cya translocation assays at 6 hpi. (B) Expression of HrpP in *Arabidopsis* partially restored the virulence of the *hrpP* mutant. Col-0 and *hrpP*-transgenic *Arabidopsis* plants were inoculated with DC3000, DC3000 Δ*hrcQ–U*, and DC3000 Δ*hrpP* at 1 × 10^5^ CFU/mL. Samples were taken at 3 dpi for bacterial counts. (C) Expression of HrpP in *Arabidopsis* suppresses the flg22-induced ROS burst. Leaf disks from Col-0 and HrpP-transgenic *Arabidopsis* plants were first soaked in water for 12 h and then subjected to an ROS burst assay as described in the legend of Fig. 1C. (D) Expression of HrpP in *Arabidopsis* suppresses flg22-induced callose deposition. Leaves were infiltrated with 1 μM flg22 and collected 15 h after treatment for aniline blue staining (bars = 100 μm). All of the experiments were repeated three times, with similar results. The data shown are the means ± standard deviations. The lowercase letters above the error bars indicate statistically significant differences among treatments (*P <* 0.05 by one-way ANOVA).

As is the case for P. syringae pv. *tomato* DC3000 Δ*hrcQ–U*, the *hrpP* mutant failed to deliver an effector-reporter hybrid (AvrPto tagged with the adenylate cyclase (Cya) translocation reporter, AvrPto-Cya), as previously reported (31) (Fig. 2A). However, it was unclear whether HrpP functions in facilitating effector delivery from outside the bacterial and plant cells. Therefore, we infiltrated HrpP proteins into the leaves of N. benthamiana and determined whether exogenous HrpP could restore the effector translocation of the *hrpP* mutant of P. syringae pv. *tomato* DC3000. The presence of HrpP in plants significantly stimulated the AvrPto-Cya translocation of P. syringae pv. *tomato* DC3000 Δ*hrpP* but not DC3000 or DC3000 Δ*hrcQ–U* (Fig. 2A). We also examined whether *hrpP*-transgenic plants affected host immunity and bacterial pathogenicity by generating transgenic *Arabidopsis* plants that constitutively expressed *hrpP* under the control of the cauliflower mosaic virus (CaMV) 35S promoter. The transgenic plants exhibited high levels of the *hrpP* transcript and the same growth phenotype as that of wild-type (WT) Col-0 (Fig. S2A and B). We then inoculated P. syringae pv. *tomato* DC3000 and the Δ*hrpP* mutant into WT and *hrpP*-transgenic *Arabidopsis* plants at 1 × 10^5^ CFU/mL. The results showed that at 3 dpi, P. syringae pv. *tomato* DC3000 had grown to a higher level in the transgenic plants than in the WT *Arabidopsis* plants (Fig. 2B). Notably, the *hrpP* mutant exhibited a population size similar to that of the P. syringae pv. *tomato* DC3000 Δ*hrcQ–U* mutant in WT *Arabidopsis*, whereas it propagated to significantly higher levels in the transgenic plant, outgrowing the P. syringae pv. *tomato* DC3000 Δ*hrcQ–U* mutant (Fig. 2B).

To assess whether the transgene of *hrpP* altered host innate immunity, we determined the ROS burst and callose deposition triggered by PAMPs. As shown in Fig. 2C and D, the flg22 epitope triggered significantly lower levels of ROS production and callose deposition in *hrpP*-transgenic *Arabidopsis* than in WT plants. Similar results were also obtained when flg22 was replaced by elf18, which is another typical PAMP (Fig. S2C and D). Collectively, these results suggest that HrpP may overcome host immunity and promote effector translocation to facilitate bacterial infection.

### HrpP is involved in innate immunity networks and hormone signaling.

**FIG 3:**
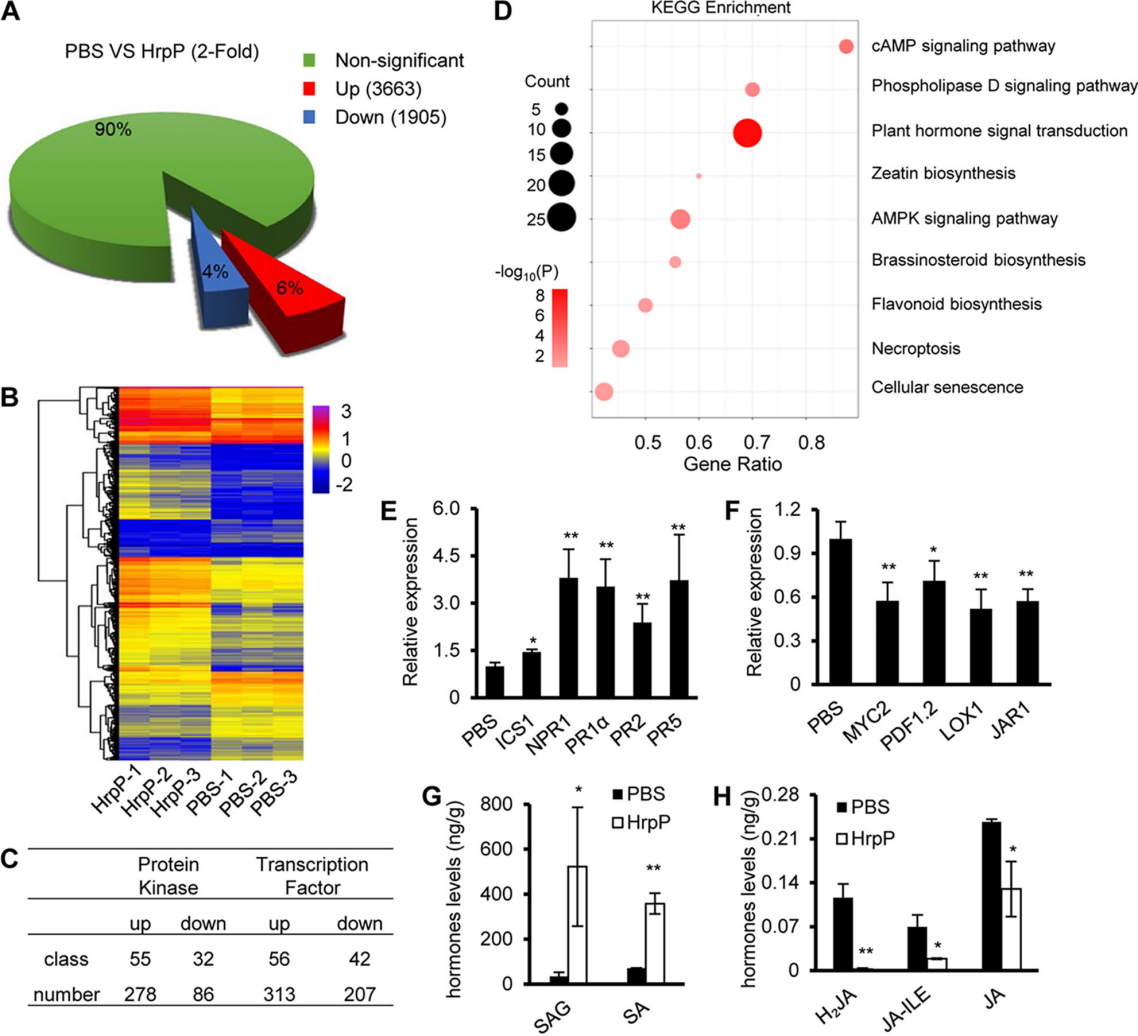
Transcriptome analysis reveals that HrpP is involved in immunity-associated kinase, TF, and hormone signaling. (A) Transcriptome overview of HrpP-regulated genes (a >2-fold change and a *P* value of <0.05) in N. benthamiana with 1 μM HrpP treatment for 6 h after infection. PBS was used as the control. (B) Heatmap of HrpP-regulated genes in N. benthamiana. Hierarchical clustering was analyzed with the average linkage method using Multiple Experiment Viewer (MeV). Colors from red to blue indicate expression levels from relatively high to low, respectively. (C) HrpP alters the expression of protein kinases and transcription factors. (D) KEGG analysis of HrpP-regulated genes in N. benthamiana. The rich factor reflects the degree of enriched differentially expressed genes (DEGs) in a given pathway. The number of enriched DEGs in the pathway is indicated by the circle area, and the circle color represents the ranges of the corrected *P* values. AMPK, AMP-activated protein kinase. (E) Relative expression of SA signaling marker genes. N. benthamiana leaves were infiltrated with PBS or 1 μM HrpP, and leaf samples were taken at 6 hpi for qRT-PCR. The *NbEF1a* gene was used as a reference gene, and data were normalized to the value for the PBS control. (F) Relative expression levels of JA signaling marker genes. The procedure was the same as the one described above. (G) Quantitative determination of SA in HrpP-treated N. benthamiana. Plant leaves were infiltrated with PBS or 1 μM HrpP and subjected to hormone extraction and quantification at 12 hpi. (H) Quantitative determination of JA in HrpP-treated N. benthamiana. The procedure was the same as the one described above. All of the experiments were repeated three times, with four biological replicates. The data shown are the means ± standard deviations. * and ** indicate statistically significant differences (by a *t* test) at *P* values of <0.05 and <0.01, respectively.

To characterize the plant response at the transcriptomic level, we performed transcriptome sequencing (RNA-seq) of N. benthamiana leaves treated with 1 μM HrpP for 6 h. An overview of the transcriptome and hierarchical clustering analyses between the phosphate-buffered saline (PBS) control and HrpP revealed that a total of 5,568 differentially expressed genes (DEGs) were regulated by HrpP sensing (representing a ≥2-fold increase [*P <* 0.05]), which represented approximately 10% of all N. benthamiana genes (Fig. 3A). We observed that a high number of genes representing ca. two-thirds of the DEGs were induced rather than suppressed (Fig. 3A and B). Functional annotation of the DEGs using MapMan revealed that HrpP was strongly involved in the biotic stress stimulus (Fig. S3A). Notably, the majority of the DEGs were related to recognition signaling, including the MAPK pathway, the regulation of transcription, secondary metabolites, and hormone signaling (Fig. S3A).

We also performed gene ontology (GO) and KEGG analyses using the N. benthamiana genome sequence as a reference (Fig. S3B and D). The “biological process” GO terms “cellular process” and “metabolic process” were the most abundant terms, and in particular, “protein phosphorylation” had the highest enrichment with respect to HrpP-regulated genes (Fig. S3B). In the “molecular function” category of GO terms, “protein kinase activity,” “transcription factor (TF) activity,” and “calcium ion binding” were the most prominent, which further highlights the relevance of protein kinases and TFs in plant immunity (Fig. S3B). Interestingly, the KEGG pathway analysis showed that most of the significant enrichments were related to plant hormone signal transduction and the biosynthesis of secondary metabolites (Fig. 3D).

Protein kinases and TFs are two well-known signaling components involved in plant immunity (3, 32–34). In our treatments, totals of 364 and 520 differentially expressed kinase and TF genes were retrieved (Fig. 3C and Fig. S4A and B). For the protein kinase genes, 278 kinase genes were induced, which were classified into 55 kinase families, including the following plant immunity-associated families: RLK-Pelle_DLSV, RLK-Pelle_RLCK-VIIa-2, STE_STE11, CAMK_CDPK, RLK-Pelle_LRR-XI-1, and RLK-Pelle_LRR-XII-1 (6, 35, 36). For the TF genes, 313 genes were induced, which fell into 56 TF families, while 207 genes were suppressed, which fell into 42 TF families. These results indicate that apoplastic HrpP can significantly alter the expression of protein kinases and TFs, which may be involved in the sensing of HrpP and HrpP-mediated plant signaling.

### HrpP alters hormone metabolism.

To verify whether HrpP mediates hormone signaling, we tested the expression of typical marker genes of salicylic acid (SA) and jasmonic acid (JA) signal transduction. The results showed that the SA-related genes *PR1a*, *PR2*, and *PR5* were significantly activated with 1 μM HrpP for 6 h in N. benthamiana (Fig. 3E). In comparison, the JA-related genes *MYC2* and *PDF1.2*, and even the biosynthesis genes *LOX1* and *JAR1*, were dramatically inhibited in the HrpP treatments (Fig. 3F). We also measured SA and JA production to clarify whether HrpP affected hormone biosynthesis. Remarkably, both free SA and inactive glycosylated SA (SAG) (37) were significantly increased in plant leaves treated with HrpP compared to the PBS control (Fig. 3G). In contrast, JA dihydrojasmonic acid (H_2_JA) and isoleucine-conjugated JA (JA-ILE), which directly bind to the JA receptor COI1 and repressor JAZ (38, 39), were significantly reduced in the same samples (Fig. 3H). These results suggest that HrpP sensing activates SA signaling and inhibits JA signaling and that this is associated with increased SA accumulation and decreased JA biosynthesis.

### HrpP physically interacts with MKK2 *in vitro* and *in vivo*.

Given our evidence that HrpP is involved in immune pathways and hormone signal transduction, we explored the potential interactors using yeast two-hybrid (Y2H) screening from an immune gene inventory, which included the well-known receptor-like kinases (FLS2, CERK1, and LORE), coreceptors (BAK1 and SOBIR1), receptor-like cytoplasmic kinases (PBL1, PBL2, PBL27, and CDPK28), MAPK cascades (MAPKKKα and MKK2), immunity regulator (SGT1), transcriptional coactivator (EDS1), and SA receptor (NPR1) (6, 36, 40–44).

**FIG 4:**
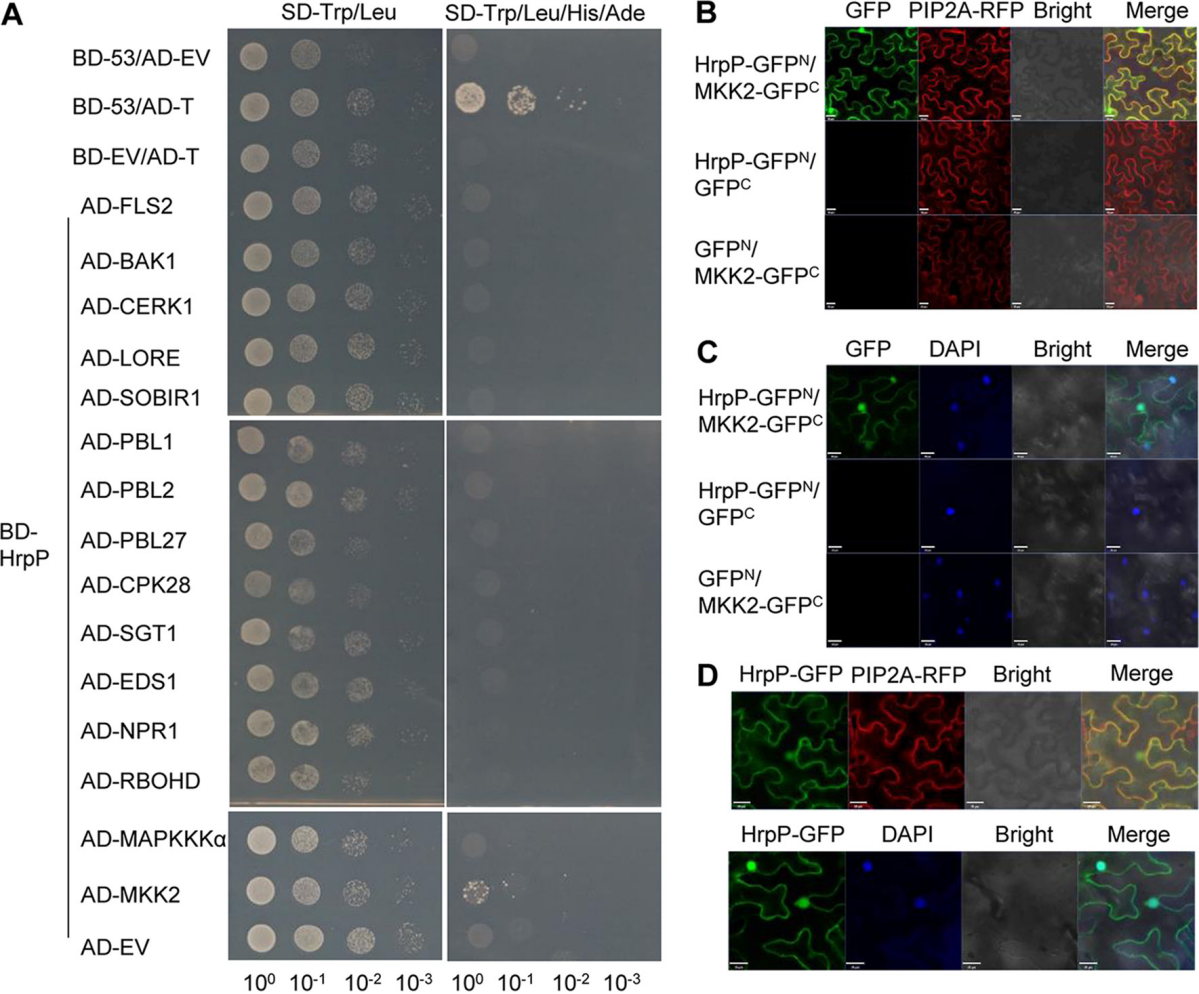
HrpP physically interacts with MKK2. (A) Interactor screening for HrpP by a Y2H assay. Yeast strain Gold with the indicated plasmid combinations was spotted in 10-fold serial dilutions onto synthetic dextrose (SD)/-Leu/Trp medium and SD/-Leu/Trp/His/Ade containing 0.01% aureobasidin A. The interaction between 53 and T served as the positive control. (B and C) HrpP interacts with MKK2 on the plant plasma membrane (PM) (B) and in the nucleus (C) in a BiFC assay. HrpP and MKK2 were fused to the N terminus (GFP^N^) and C terminus (GFP^C^) of GFP, respectively. Different combinations were coexpressed in N. benthamiana leaves by *Agrobacterium* infiltration. PIP2A-RFP was used as the PM marker, and 4′,6-diamidino-2-phenylindole (DAPI) was used to stain nuclei. Fluorescence was detected by confocal microscopy at 2 dpi. Bars = 20 μm. (D) Subcellular localization of HrpP in N. benthamiana. The HrpP-GFP fusion protein was expressed in N. benthamiana leaves by *Agrobacterium* infiltration. PIP2A-RFP was used as the PM marker, and DAPI was used to stain nuclei. Fluorescence was detected by confocal microscopy at 2 dpi. Bars = 20 μm. All experiments were repeated three times, with similar results. Leu, leucine; Trp, tryptophan; His, histidine; Ade, adenine.

The results indicated that only MKK2, the key module of the MAPK pathway, directly interacted with HrpP in yeast (Fig. 4A). We further employed bimolecular fluorescence complementation (BiFC) assays *in planta* to confirm the interaction. HrpP was fused with the N-terminal region of green fluorescent protein (GFP^N^), whereas MKK2 was fused with the C terminus (GFP^C^). The coexpression of HrpP-GFP^N^ and MKK2-GFP^C^ in N. benthamiana by *Agrobacterium*-mediated infiltration resulted in strong green fluorescence (Fig. 4B and C). We also investigated the subcellular localization of the HrpP-MKK2 interaction in plants. Using 4′,6-diamidino-2-phenylindole (DAPI) staining and the plasma membrane (PM) marker plasma membrane intrinsic protein 2A (PIP2A) tagged with red fluorescent protein (PIP2A-RFP), we confirmed that the coexpression of HrpP-GFP^N^ and MKK2-GFP^C^ was localized on the plasma membrane (Fig. 4B) and in the nucleus (Fig. 4C) of N. benthamiana. In contrast, none of the negative controls showed GFP expression. Further determination of the subcellular localization of HrpP and MKK2 showed that both were associated with the PM and the nucleus (Fig. 4D and Fig. S6), which was consistent with the localization of the HrpP-MKK2 interaction (Fig. 4B and C and Fig. S6). These results indicate that HrpP can directly interact with MKK2 *in vitro* and *in vivo* and that the formation of the HrpP-MKK2 complex occurs primarily on the PM and in the nucleus of plant cells.

### N-terminal HrpP_1–119_ is required for MKK2 interactions and immune responses in plants.

**FIG 5:**
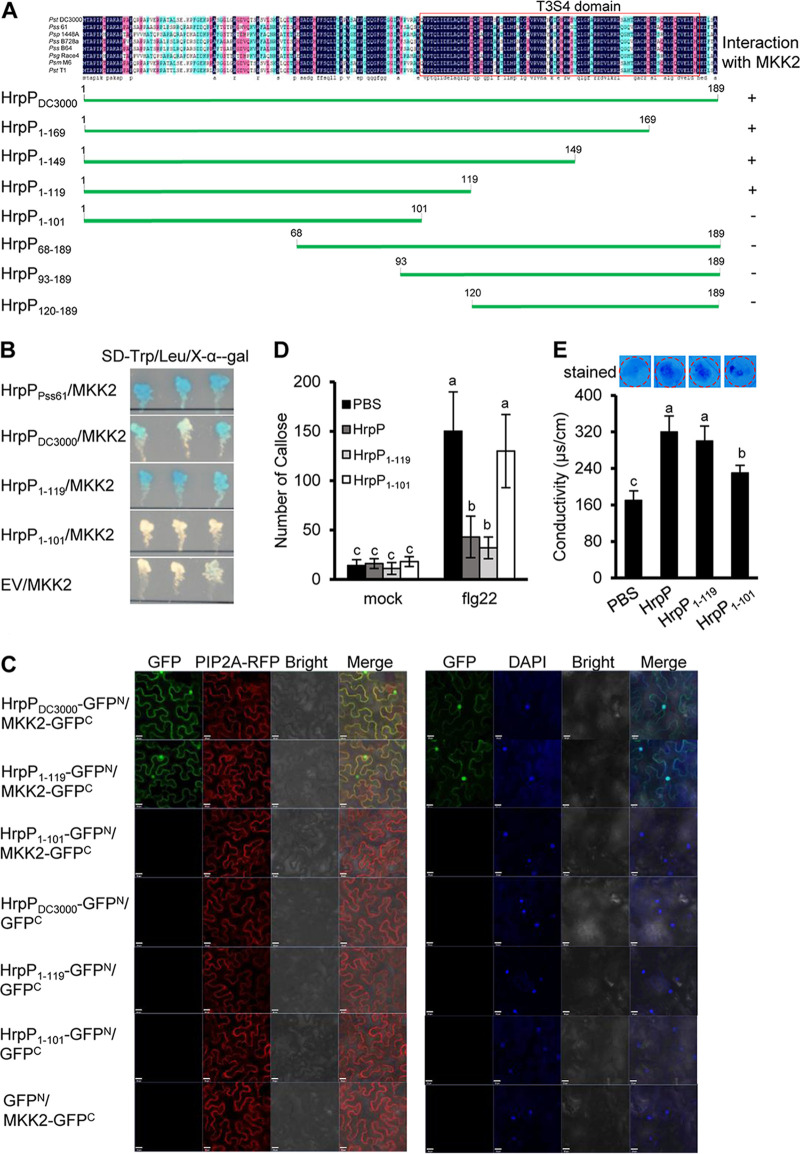
HrpP_1–119_ interacts with MKK2 and inhibits PTI. (A) Schematic diagram of the Y2H assay that pinpoints the minimal region of HrpP interacting with MKK2. HrpP truncations were cloned for a Y2H test as described in the legend of Fig. 4A. “+” represents a positive interaction, and “−” represents a negative interaction. The T3S4 domain is indicated by the red box. (B) Full-length P. syringae pv. *syringae* 61 HrpP and the P. syringae pv. *tomato* DC3000 HrpP_1–119_ truncation interact with MKK2 in a Y2H assay. (C) HrpP_1–119_ interacts with MKK2 on the plant plasma membrane and in the nucleus in a BiFC assay, as described in the legend of Fig. 4B and C. (D) HrpP_1–119_ was sufficient to inhibit flg22-induced callose deposition, as described in the legend of Fig. 1E. (E) HrpP_1–101_ elicits plant cell death, as described in the legend of Fig. 1A and B. All of the experiments were repeated three times, with similar results. The data shown are the means ± standard deviations. The lowercase letters above the error bars indicate statistically significant differences among treatments (*P < *0.05 by one-way ANOVA). Leu, leucine; Trp, tryptophan; X-α-gal, 5-bromo-4-chloro-3-indolyl-α-d-galactopyranoside.

As previously noted, HrpP is a conserved T3SS protein among P. syringae pathovars, including P. syringae pv. *syringae* 61, with some unannotated conserved regions (45) (Fig. 5A). To investigate the universality of the HrpP-MKK2 interaction, we cloned the full-length *hrpP* gene from P. syringae pv. *syringae* 61 to test the interaction with MKK2. The Y2H assay showed that the HrpP protein of P. syringae pv. *syringae* 61 was directly associated with MKK2 (Fig. 5B). Subsequently, we determined and mapped the region of the HrpP protein that was essential for the interaction with MKK2 using a series of truncated versions of the protein according to the conserved regions (Fig. 5A). Notably, the N-terminal region of the HrpP protein proved essential for the interaction with MKK2, whereas none of the C-terminal truncations showed binding activity (Fig. 5A and Fig. S5). Furthermore, both the Y2H (Fig. 5B) and BiFC (Fig. 5C and Fig. S7) assays showed that the N terminus of HrpP_1–119_ interacted with MKK2 *in vitro* and *in vivo*, while the shorter HrpP_1–101_ protein lost the binding ability. The subcellular localization assays showed that HrpP_1–119_ was associated with MKK2 on the PM and in the nucleus of the plant cells (Fig. 5C).

**FIG 6:**
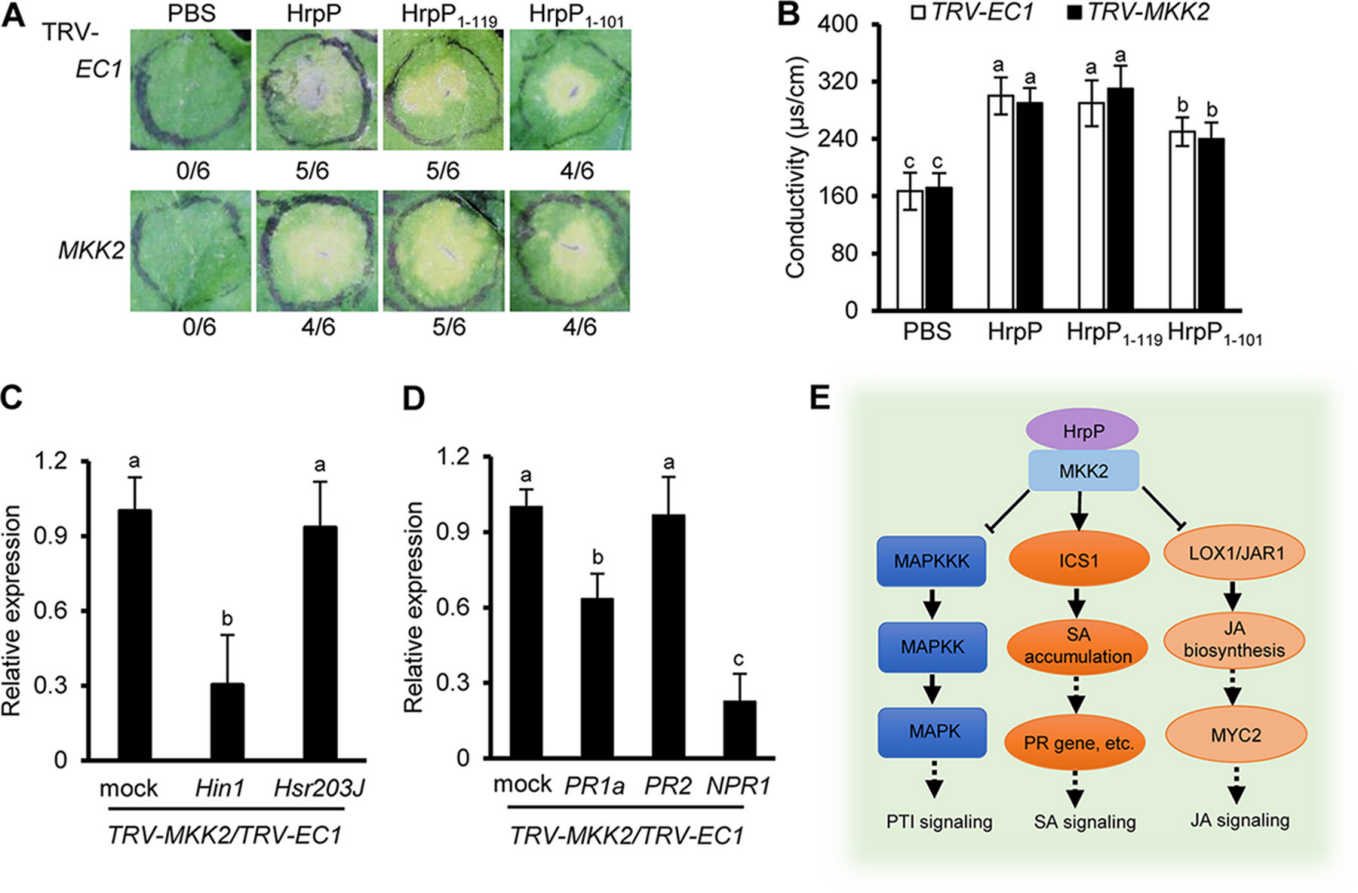
Model for the role of HrpP in bacterial pathogenesis. (A) HrpP induces MKK2-independent cell death. MKK2-silenced N. benthamiana plants were infiltrated with PBS or different HrpP derivatives at 20 μM for cell death assessment at 2 dpi. EC1 was used as a control. The fraction below each image indicates the number of times that the HR was present relative to the number of test inoculations. (B) Conductivity was assayed at 1 dpi. (C) Relative expression levels of cell death marker genes in MKK2-silenced N. benthamiana plants. EC1- and MKK2-silenced N. benthamiana plants were infiltrated with PBS or 1 μM HrpP, and leaf samples were taken at 6 hpi for qRT-PCR. The *NbEF1a* gene was used as a reference gene, and data were normalized to the values for the negative control. (D) Relative expression levels of SA signaling marker genes in MKK2-silenced N. benthamiana plants. The procedure was the same as the one described above. (F) HrpP interacts with MKK2 on the plant plasma membrane or in the nucleus to suppress PTI. MKK2 is required for plant innate immunity suppression and the induction of SA signaling. HrpP sensing induces SA accumulation, suppresses JA accumulation, and promotes effector translocation and bacterial infection. The data shown are the means ± standard deviations. The lowercase letters above the error bars indicate statistically significant differences among treatments (*P < *0.05 by one-way ANOVA). All of the experiments were repeated three times, with similar results.

We also examined whether the region spanning residues 1 to 119 of the HrpP protein was involved in PTI suppression and cell death elicitation. For this, we expressed and purified His-tagged HrpP_1–119_ and HrpP_1–101_ proteins from Escherichia coli strain BL21(DE3) (Fig. S1A) and infiltrated this mixture into the leaves of N. benthamiana to test callose deposition and cell death. As with the full-length HrpP protein, the HrpP_1–119_ truncation substantially inhibited flg22-induced callose deposition (Fig. 5D), whereas HrpP_1–101_ did not suppress callose deposition (Fig. 5D). In contrast, both the HrpP_1–119_ and HrpP_1–101_ proteins were able to induce cell death and cause electrolyte leakage in N. benthamiana, although the cell death triggered by HrpP_1–101_ was moderate compared to that triggered by HrpP_1–119_ (Fig. 5E). MKK2 is a common MAPK protein involved in PTI signaling (46). Therefore, given the differences in PTI suppression and cell death elicitation between HrpP_1–119_ and HrpP_1–101_ and the fact that only HrpP_1–119_ interacts with MKK2, we infer that MKK2 may not mediate HrpP-triggered cell death. To confirm this, we used virus-induced gene silencing (VIGS) to knock down *MKK2* gene expression in N. benthamiana (Fig. S8) and tested whether the HrpP protein elicited cell death. Remarkably, both full-length HrpP and the two truncations (HrpP_1–119_ and HrpP_1–101_) continued to trigger cell death and cause electrolyte leakage in *MKK2*-silenced plants to the same extent as the EC1 control (Fig. 6A and B). Further tests showed that *MKK2* silencing repressed the transcript accumulation of the cell death marker gene *Hin1* but not *Hsr203J* (Fig. 6C). Also, *MKK2* silencing impaired the HrpP-mediated induction of *PR1a* and *NPR1* (Fig. 6D). Collectively, these findings indicate that the HrpP-MKK2 interaction is associated with PTI suppression and that MKK2 is required for HrpP-induced SA signaling in N. benthamiana.

## DISCUSSION

Most of the genes of the T3SS cluster are required for T3SS assembly and effector delivery (15); however, few studies have been conducted on the T3SS Hrp protein mechanisms in plant-pathogen interactions. Previous research has shown that some T3SS components, such as harpins, can be secreted or translocated into plant cells, and the exogenous application of harpin proteins *in planta* can elicit an ETI-like response (20, 22, 47). Our work shows that the T3SS protein HrpP can interact with MKK2, resulting in PTI suppression. We also demonstrate that HrpP can induce plant cell death in the absence of MKK2 interactions. Furthermore, HrpP promotes bacterial effector translocation and virulence *in vitro* and in transgenic plants, probably by sensing HrpP directly or interacting with T3SS components or the translocon. We noticed that apoplastic HrpP cannot fully restore the ability to translocate the effector of the *hrpP* mutant, which suggested that apoplastic HrpP and HrpP inside bacterial cells may work cooperatively to manipulate T3SS function (31). To the best of our knowledge, this is the first report of a T3SS Hrp protein being involved in the feedback control of T3SS associations with plant cells and the targeting of the MAPK cascade to suppress plant innate immunity. These findings highlight an important battleground in plant-microbe interactions by demonstrating a novel virulence mechanism associated with the P. syringae T3SS.

MAPK cascades, which are hot targets for pathogenic bacterial type III effectors (T3Es), play a critical role in regulating plant immunity (48–52). For example, the P. syringae effector HopF2 suppresses PTI by targeting MKK5, most likely through its ADP-ribosyltransferase activity (49). HopAI1 perturbs the MEKK1-MKK5-MPK3/MPK6 pathway by inactivating MPK3/6 proteins via its phosphothreonine lyase activity (48). HopZ1a acetylates the positive defense regulator MKK7, suppressing MKK7-dependent responses in *Arabidopsis* (51). Except for the T3S4 domain, no more obvious domain was observed in the HrpP protein. However, our results showed that the HrpP-MKK2 interaction relies on N-terminal amino acids (aa) 101 to 119 of HrpP but not the C-terminal T3S4 domain (Fig. 5D), in which the last 20 to 40 aa are crucial for effector secretion and translocation. Using NetPhos3.1 and GPS5.0, we examined the region spanning aa 101 to 119 and discovered an serine/threonine kinase (STE) family kinase phosphorylation site at threonine 104. It remains to be determined whether this site is required for MKK2 interactions. However, our findings that the silencing of *MKK2* reduced the HrpP-mediated induction of SA signaling (Fig. 6D) suggested that HrpP may rely on MKK2 to modulate SA signaling. Similarly, another study found that after Botrytis cinerea infection, the expression of the SA signaling marker gene was significantly reduced in MKK2-silenced tomato plants (53). In *Arabidopsis*, knocking down AtMKK4 and AtMKK5 also compromised the expression of SA signaling marker genes after P. syringae infection (54). In addition, Hu et al. showed previously that the geminivirus βC1 protein, which lacks enzymatic activity, interacts with MKK2 and inhibits the kinase activity of MKK2 in phosphorylating MPK4 (55). As a result, it is necessary to investigate whether the HrpP protein inactivates MKK2 kinase activity and, thus, quells plant immunity by directly binding to MKK2 and its active sites. We determined the subcellular localization of HrpP and MKK2 to confirm their interaction. Both HrpP and MKK2 can be found on the plasma membrane and in the nucleus (Fig. 4D; see also Fig. S6 in the supplemental material), which is consistent with the location of the HrpP-MKK2 interaction (Fig. 4B and C and Fig. S6). Additionally, it was shown that SaMKK2 was associated with the cytoplasm and nucleus of Solanum acaule ACL-27 (56). Although we do not know whether the location of MKK2 varies among plant species, these findings suggest that MKK2 may function in multiple plant compartments.

Although the interaction of HrpP with MKK2 is required for PTI suppression, MKK2 silencing did not block HrpP-triggered cell death completely in N. benthamiana. This implies that other receptors are possibly essential for cell death. Given that HrpP can be translocated into plant cells via the T3SS, we silenced a number of known immunity proteins involved in intracellular cell death sensing and downstream signaling to identify these potential factors. Usually, SGT1 (a suppressor of the G2 allele of *skp1*) is associated with RAR1 (required for Mla12 resistance) and HSP90 (heat shock protein 90), forming a complex that manipulates nucleocytoplasmic nucleotide-binding/leucine-rich-repeat (NLR) proteins for effector perception (43, 57). Another ETI regulator, enhanced disease susceptibility 1 (EDS1), is a conserved lipase-like protein that forms a dimer with either PAD4 or SAG101 to mediate cell death triggered by plant NLRs (58, 59). It has been reported that the silencing of either MAPKKKα, salicylate-induced protein kinase (SIPK), or wound-induced protein kinase (WIPK) compromises R gene-mediated resistance to viral and bacterial pathogens (41, 60, 61); however, we found that knocking down SGT1, RAR1, and EDS1 or MAPKKKα, SIPK, and WIPK did not result in significantly reduced HrpP-induced cell death (Fig. S8). These results imply possible apoplastic HrpP-induced cell death sensing at the plasma membrane rather than cytoplastic effector-induced cell death recognition (62, 63). Several studies suggest that most of the invasion patterns characterized to date are recognized by proteinaceous PRRs (64), yet some elicitins and T3SS components, such as HrpZ1 and HrpN, may directly interact with plant lipids (and not PRRs), either modulating the plasma membrane or using lipid decoration as the receptor/target to trigger cell death (65). Although we have not determined how the T3SS protein HrpP interacts with the plant plasma membrane, we assume that HrpP might be recognized as a “danger” signal that activates strong innate immune responses, such as *PR1* expression, SA accumulation, and active plant cell death. This is partially supported by the findings that P. syringae infection stimulates SA-related marker gene expression and SA accumulation in tomato and *Arabidopsis* plants (66, 67).

Based on our observations, we propose a model for the role of HrpP in bacterial pathogenesis (Fig. 6C). In this model, HrpP is secreted in the apoplast and is translocated into plant cells through the P. syringae T3SS. Apoplastic HrpP proteins, even the short truncation HrpP_1–101_, induce cell death-like immunity through an unknown mechanism, whereas HrpP and the truncation HrpP_1–119_ interact with MKK2 on the plant plasma membrane or in the nucleus to suppress PTI. MKK2 is required for plant innate immunity suppression but is dispensable for cell death caused by the HrpP proteins. This highlights the divergence of HrpP in cell death induction and PTI suppression. HrpP sensing alters plant transcriptomic expression, resulting in increased SA accumulation and decreased JA accumulation, as well as promoting effector translocation and bacterial infection. Overall, our study reveals that the P. syringae T3SS protein HrpP is involved in the fine-tuning of plant immunity. Further studies on the molecular and biochemistry mechanisms of HrpP sensing will shed much-needed light on the roles of T3SS components in plant-microbe interactions.

## MATERIALS AND METHODS

### Strains, primers, and plasmids.

Strains, primers, and plasmids used in this study are listed in Tables S1 to S3 in the supplemental material, respectively. All Pseudomonas syringae strains were grown on King’s B (KB) medium with the appropriate antibiotics at 28°C. Agrobacterium tumefaciens and Escherichia coli were grown on Luria-Bertani (LB) medium with the appropriate antibiotics at 28°C and 37°C, respectively. The following antibiotics were used: ampicillin (100 μg/mL), kanamycin (50 μg/mL), rifampicin (50 μg/mL), and spectinomycin (50 μg/mL).

### Plant material and pathogen infection assay.

N. benthamiana and S. lycopersicum plants were grown in a controlled environmental chamber at 25°C with a 16-h-light/8-h-dark photoperiod. Arabidopsis thaliana plants were grown at 23°C with a 10-h-light/14-h-dark photoperiod. Unless otherwise stated, 4- to 5-week-old plants were used for all experiments. For the bacterial infection assay, *Arabidopsis* plants were inoculated with P. syringae at 1 × 10^5^ CFU/mL, and plants were covered with a plastic lid to maintain high humidity for 1 day. Leaf disks collected at the indicated time points were processed with 200 μL of KB medium, serially diluted, and spotted onto KB plates supplemented with the appropriate antibiotics for bacterial counts.

### Generation of transgenic *Arabidopsis* plants.

For *hrpP* overexpression in *Arabidopsis*, the *hrpP* genomic sequence was cloned into the binary vector pB7WG2.0 (Invitrogen, USA) controlled by the CaMV 35S promoter with a Flag epitope tag. The transformation of *Arabidopsis* Col-0 was performed by the floral dip method (4) using A. tumefaciens strain GV3101 harboring the indicated vector. The transgenic lines were screened by Basta and immunoblot analysis with anti-Flag antibody (catalog number M185-7; MBL, Japan).

### Purification of recombinant proteins.

E. coli strain BL21(DE3) harboring an expression vector was grown in 2 L of LB medium and induced with 1 mM isopropyl β-d-thiogalactoside at an optical density at 600 nm (OD_600_) of 0.6 to 0.8 at 16°C for 18 h. The proteins were purified by affinity chromatography using a His-Trap HP column (GenScript, USA) as previously described (68). Purified proteins were dialyzed overnight into phosphate-buffered saline (PBS) (137 mM NaCl, 2.7 mM KCl, 10 mM Na_2_HPO_4_, 2 mM KH_2_PO_4_ [pH 7.4]), concentrated to 100 μM, and then stored at −80°C after filtration sterilization using a 0.22-μm filter.

### Cya translocation reporter assays.

Translocation assays were performed as previously described, with minor modifications (69). Briefly, N. benthamiana leaves were infiltrated with PBS or 1 μM HrpP protein. The infiltrated area was challenged 6 h later by the inoculation of bacterial strains carrying plasmids expressing AvrPto-Cya at 1 × 10^7^ CFU/mL. Six hours after the challenge inoculation, leaf disks were collected, and cAMP levels were determined by using a Correlate-EIA cAMP immunoassay kit according to the manufacturer’s instructions (catalog number ADI-901-066; Enzo, USA).

### Conductivity detection.

Cell death progression was assayed by measuring ion leakage as previously described, with minor modifications (70). Briefly, each sample contained six leaf disks collected from three different N. benthamiana leaves using a 1-cm-diameter cork borer 1 day after infiltration and then immersed in 4 mL of deionized water in a tissue culture plate. After shaking at 220 rpm for 3 h at 28°C, the conductivity of the water was measured using a conductivity meter (S230; Seven Compact, China).

### Trypan blue staining.

N. benthamiana leaves infiltrated with PBS or HrpP proteins were sampled at 1 dpi. Whole leaves were boiled for 5 min in a 1:1 mixture of 95% ethanol and trypan blue staining solution (10 mL lactic acid, 10 mL phenol, 10 mL glycerol, and 10 mg trypan blue, dissolved in 10 mL distilled water) and then destained in chloral hydrate (250 g chloral hydrate dissolved in 100 mL distilled water) for observation.

### ROS assay.

Analysis of ROS production in *Arabidopsis* plants was performed as previously described (71). For the ROS assay in N. benthamiana and *S. lycopersicum* plants, leaves were infiltrated with PBS or 1 μM HrpP protein. At 6 h postinoculation (hpi), leaf disks were collected, soaked in water for 12 h, and then transferred to a solution containing 34 mg/mL of luminol, 10 μg/mL of horseradish peroxidase, and 1 μM flg22. Luminescence was subsequently measured using a 96-well microplate luminometer plate reader (infinite M200 Pro; Tecan, China).

### Callose deposition assay.

Callose deposition assays in *Arabidopsis* plants were performed as previously described (72). N. benthamiana plant leaves were infiltrated with PBS or 1 μM HrpP protein. At 6 hpi, the inoculated leaves were challenged with 1 μM flg22. After 15 h, the collected leaves were used for aniline blue staining and observed using confocal laser scanning microscopy (LSM 880; Carl Zeiss, Germany).

### Y2H assay.

Y2H assays were performed as previously described, with minor modifications (73). Briefly, the *hrpP* gene was inserted into pGBKT7 using a seamless clone (CL116; Biomed, China) as the bait, and the indicated candidate genes were cloned into pGADT7 using a seamless clone (CL116; Biomed, China) as the prey. Cotransformed yeast cells were screened on SD/-Leu/-Trp plates. The interaction between HrpP and candidate proteins was subsequently tested on SD/-His/-Leu/-Trp/-Ade or SD/-His/-Leu/X-α-Gal plates.

### Subcellular localization.

The *hrpP* gene was cloned into the binary vector pEarlyGateS101 to make C-terminal GFP fusions and transiently expressed in N. benthamiana according to methods in a previous report (69). The RFP-fused PM marker PIP2A-RFP and the fluorescent nuclear dye DAPI (catalog number SL7101; Coolaber, China) were used for colocalization assays. After 2 dpi, leaf disks were taken from the infiltrated leaves, and subcellular localization assays were performed using confocal laser scanning microscopy (LSM 880; Carl Zeiss, Germany) with the following excitation wavelengths: 488 nm for GFP, 561 nm for RFP, and 405 nm for DAPI.

### BiFC assay.

BiFC assays were performed as previously described (74). Briefly, the HrpP protein and truncated HrpP proteins were fused to the N-terminal fragment of GFP, and the MKK2 protein was fused to the C-terminal fragment of GFP. The protein pairs fused with the indicated tags were coexpressed in N. benthamiana. Leaf disks were taken from the infiltrated leaves at 2 dpi, and images were captured using confocal laser scanning microscopy (LSM 880; Carl Zeiss, Germany) as described above.

### RNA isolation and quantitative real-time PCR assay.

N. benthamiana leaves infiltrated with PBS or the HrpP protein were collected from infiltrated areas at 6 hpi and ground in liquid nitrogen. Total RNA was isolated using a plant RNA 425 kit (catalog number R6827-02; Omega, USA) according to the manufacturer’s instructions. The FastKing RT kit (catalog number KR116-02; Tiangen, China) was used to remove genomic DNA and synthesize the first-strand cDNA. Tenfold-diluted solutions of the reaction products were used for the quantitative real-time PCR (qRT-PCR) assay. qRT-PCR was performed in a 12-μL volume with Bestar SYBR green quantitative PCR (qPCR) master mix (catalog number AG11701; Accurate Biology, China) on the ABI Quant Studio 6 Flex system (Thermo Fisher, USA).

### RNA-seq analysis.

Six leaf disks were collected from three different N. benthamiana leaves after infiltration with PBS or 1 μM HrpP protein for 6 h and immediately frozen in liquid nitrogen. Thereafter, the leaf disks were sent to Genewiz Ltd., China, for library construction. The Illumina NovaSeq 6000 platform was used for transcriptome sequencing. Reads of the raw RNA-seq data were filtered using Cutadapt (version 1.9.1). To measure gene expression levels, the abundance of each gene was normalized to fragments per kilobases per million mapped fragments (FPKM). DESeq2 (version 1.6.3) software was applied to identify differentially expressed genes (DEGs). Log_2_ fold change (log_2_FC) values (|log_2_FC| of ≥1) and *P* values (*P* < 0.05) were used as statistical significance indices. GO (http://www.geneontology.org/) enrichment analysis of the DEGs was performed using the topGO (version 2.42.0) R package. GO terms with a *P* value of <0.05 were considered significantly enriched. KEGG (http://www.genome.jp/kegg/) enrichment analysis was then performed to identify the statistically significant enrichment of DEGs in the KEGG pathways with a *P* value of <0.05. Additionally, the MapMan (version 3.5.1) package was employed to derive a graphical representation of the DEGs that played a role in the biotic stress response and metabolic pathways.

### Detection of phytohormones.

Approximately 0.3 g of N. benthamiana leaves was collected after infiltration with PBS or 1 μM HrpP at 12 hpi and immediately frozen in liquid nitrogen. The extraction and quantification of SA and JA were conducted according to the manufacturer’s protocol (Wuhan MetWare Biotechnology Co. Ltd., China). The phytohormone content was detected using MetWare (http://www.metware.cn/) based on the AB Sciex Qtrap 6500 liquid chromatography-tandem mass spectrometry (LC-MS/MS) platform.

### VIGS assay.

VIGS assays were performed as previously described (75). Briefly, 2-week-old WT N. benthamiana plants were inoculated with A. tumefaciens GV3101 carrying the helper plasmid pTRV1 mixed 1:1 with a strain carrying the pTRV2 plasmid harboring the indicated gene. Two weeks later, qPCR was used to confirm the silencing efficacy, and the silenced leaves were used for protein inoculation assays.

### Data availability.

The raw RNA-seq data are available in the NCBI database under BioProject accession number PRJNA857364. Data analysis was performed using Microsoft Excel by Student’s *t* test and one-way analysis of variance (ANOVA). *P* values of <0.05 (indicated by * in the figures) and <0.01 (**) were considered statistically significant. All quantitative data are presented as means ± standard deviations (SD).
