# Supplementary material for: Pseudomonas syringae Type III Secretion Protein HrpP Manipulates Plant Immunity To Promote Infection
Source: Microbiol Spectr. 2023 Apr 17;11(3):e05148-22. doi: 10.1128/spectrum.05148-22 (PMC10269811; doi:10.1128/spectrum.05148-22)
Supplement: Supplemental file 1 — Fig. S1 to S8. Download spectrum.05148-22-s0001.pdf, PDF file, 0.6 MB [file spectrum.05148-22-s0001.pdf]

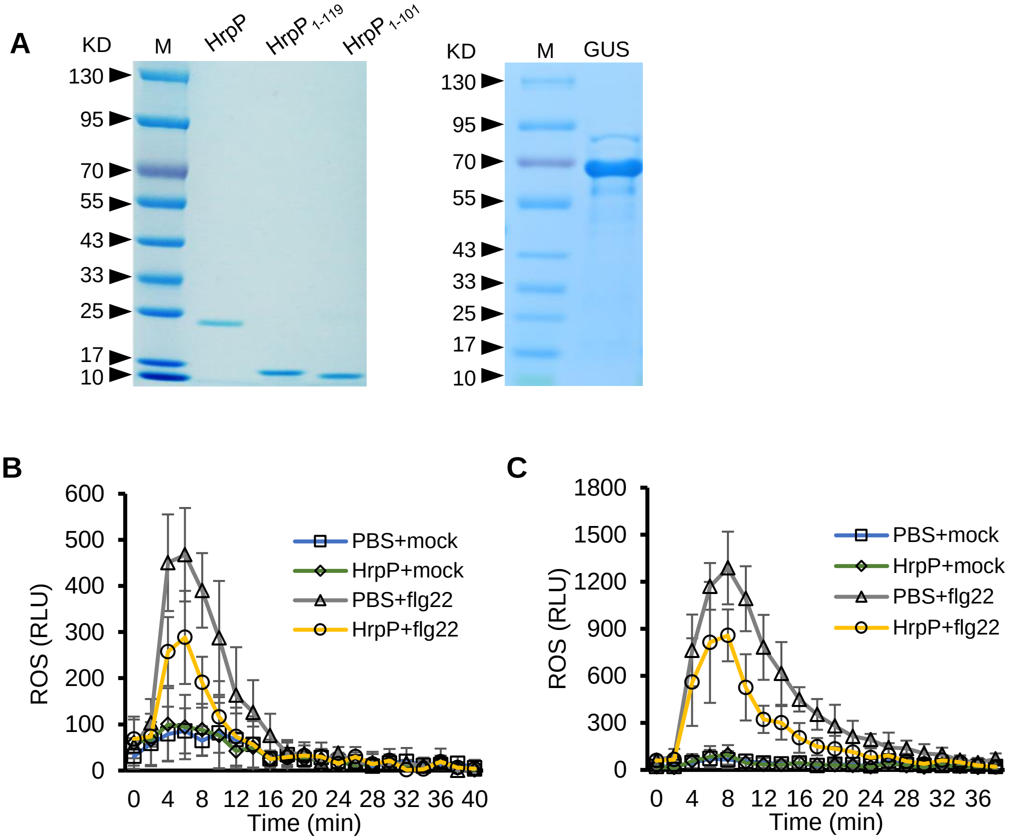

FIG S1 Purification of HrpP protein and characterization of ROS suppression ability in tomato and *Arabidopsis*.

(A) SDS–PAGE analysis of purified GUS, HrpP and truncated proteins.

(B, C) Purified HrpP protein inhibited flg22-induced ROS burst in *Solanum lycopersicum* (B) and *Arabidopsis* (C). *Solanum lycopersicum* and *Arabidopsis* leaves were infiltrated with PBS or 1 μM HrpP protein. At 6 hpi leaf discs were first soaked in water for 12 hours and then subject to ROS burst assay as described in Figure 1D.

All experiments were repeated at least three times and similar results were observed. Data shown are means ± standard deviation.

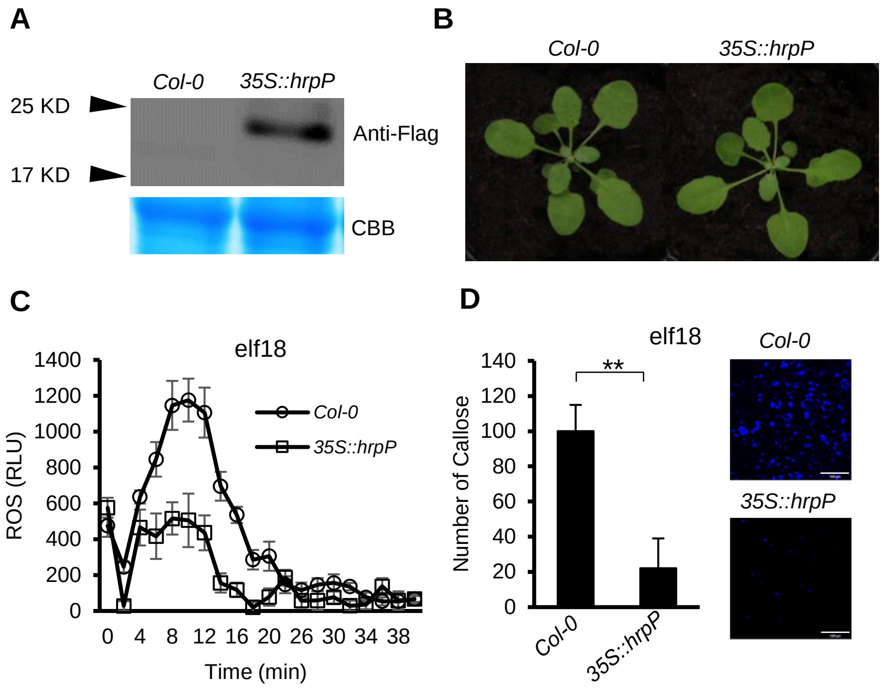

FIG S2 Transgenic expression of *hrpP* in *Arabidopsis* and determination of PTI suppression triggered by elf18.

(A) HrpP protein abundance in HrpP-overexpression transgenic plant was detected by immunoblot analysis with anti-Flag antibody. Total protein was extracted from 4-week-old seedlings and separated by SDS-PAGE. Coomassie brilliant blue (CBB) staining of plant total proteins was shown as a loading control.

(B) Transgenic *Arabidopsis* plant overexpressing *hrpP* displayed a wild-type-like growth phenotype. Four-week-old plants grown under standard short-day growth conditions were photographed.

(C, D) Expression of HrpP in *Arabidopsis* suppressed elf18-induced ROS burst (C) and callose deposition (D). ROS accumulation and callose deposition were detected as described in Figure 2C and 2D, respectively, in which flg22 was replaced elf18.

All experiments were repeated at least three times and similar results were observed. Data shown are means ± standard deviation. ** indicates the statistically significant difference (*t*-test) at *P*<0.01.

A

B

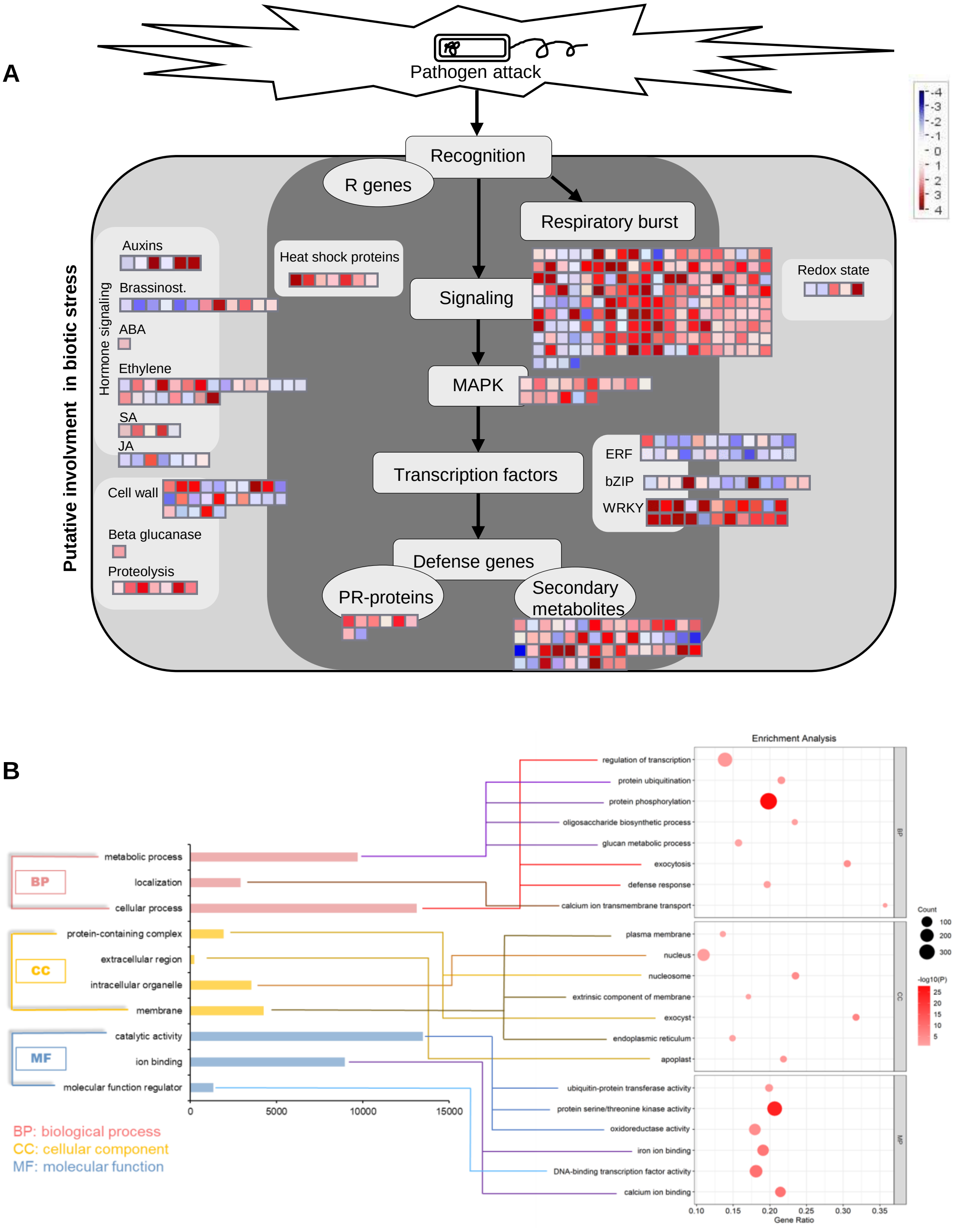

FIG S3 MapMan and GO analysis of DEGs influenced by HrpP infiltration in *N. benthamiana*.

(A) MapMan analysis of HrpP-regulated genes in *N. benthamiana*. Each gene involved in biotic stress pathway is depicted by color signal where red signifies the genes expressed highly and green indicates the genes downregulated in PBS vs HrpP. The intensity of the color is represents the level of expression.

(B) Gene Ontology (GO) terms of the HrpP-regulated genes in *N. benthamiana*. DEGs regulated by HrpP were submitted to the ShinyGO v0.60 Gene Ontology Enrichment Analysis tool (http://bioinformatics.sdstate.edu/go/) using default parameters. A Fisher's exact test was applied to identify most enriched GO terms in Biological Process (BP), Molecular Function (MF), and Cellular Component (CC) categories.

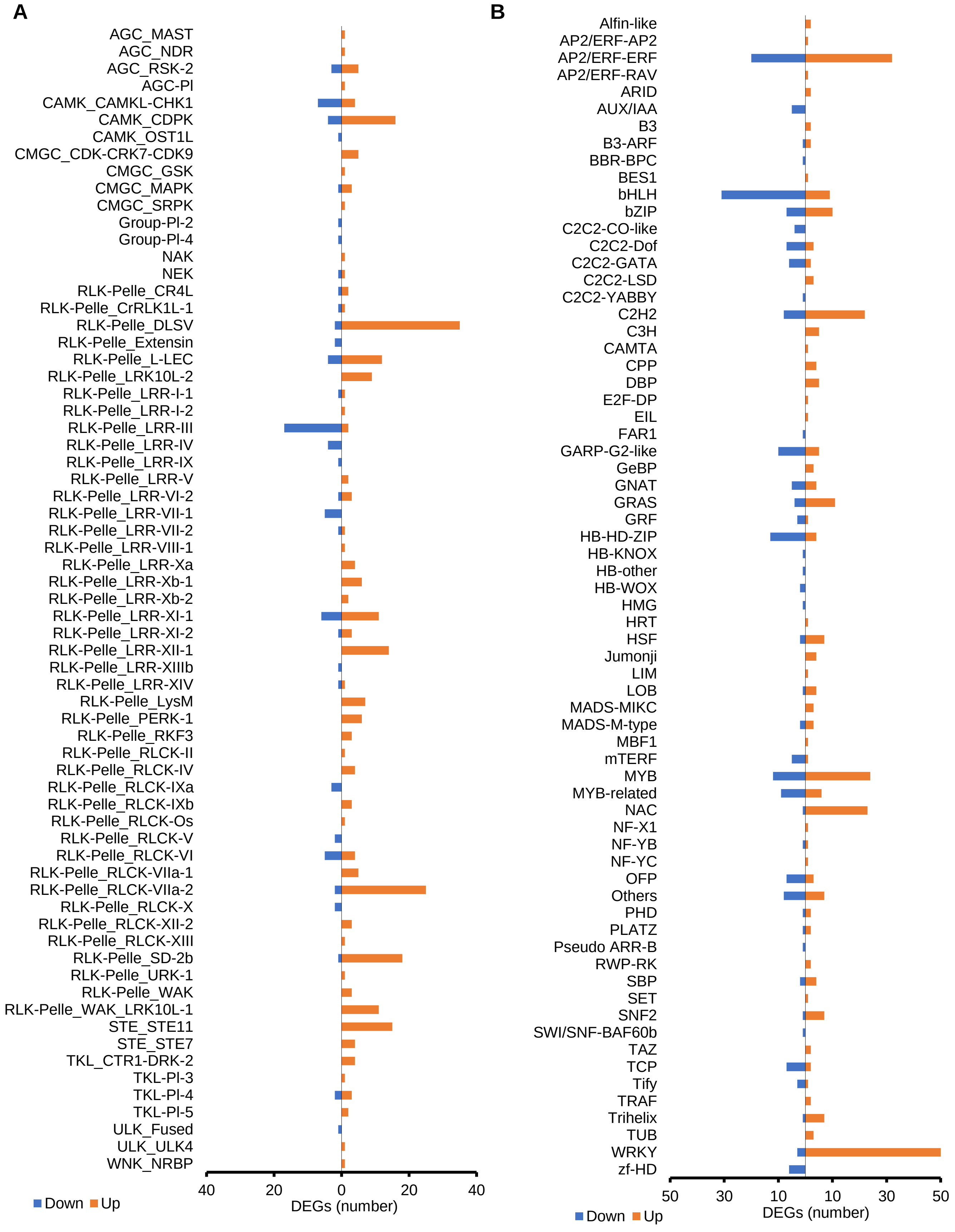

FIG S4 Protein kinase (A) and transcript factor (B) affected by HrpP infiltration in *N. banthamiana.*

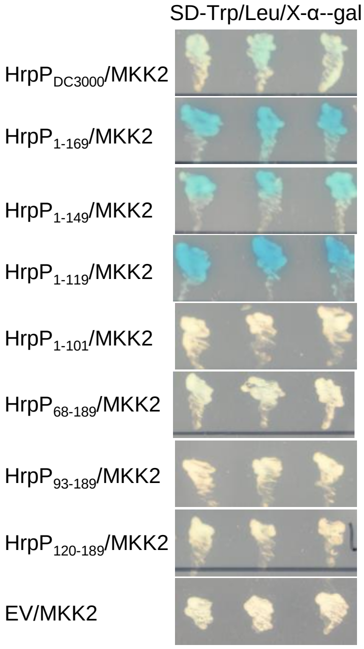

FIG S5 Full length of the Pst DC3000 HrpP and HrpP$_{1–119}$ interacts with MKK2 in a Y2H assay. HrpP truncations were cloned for a Y2H test as described in Fig. 5A. The strains covered with blue color represent positive interaction and white color represent negative interaction.

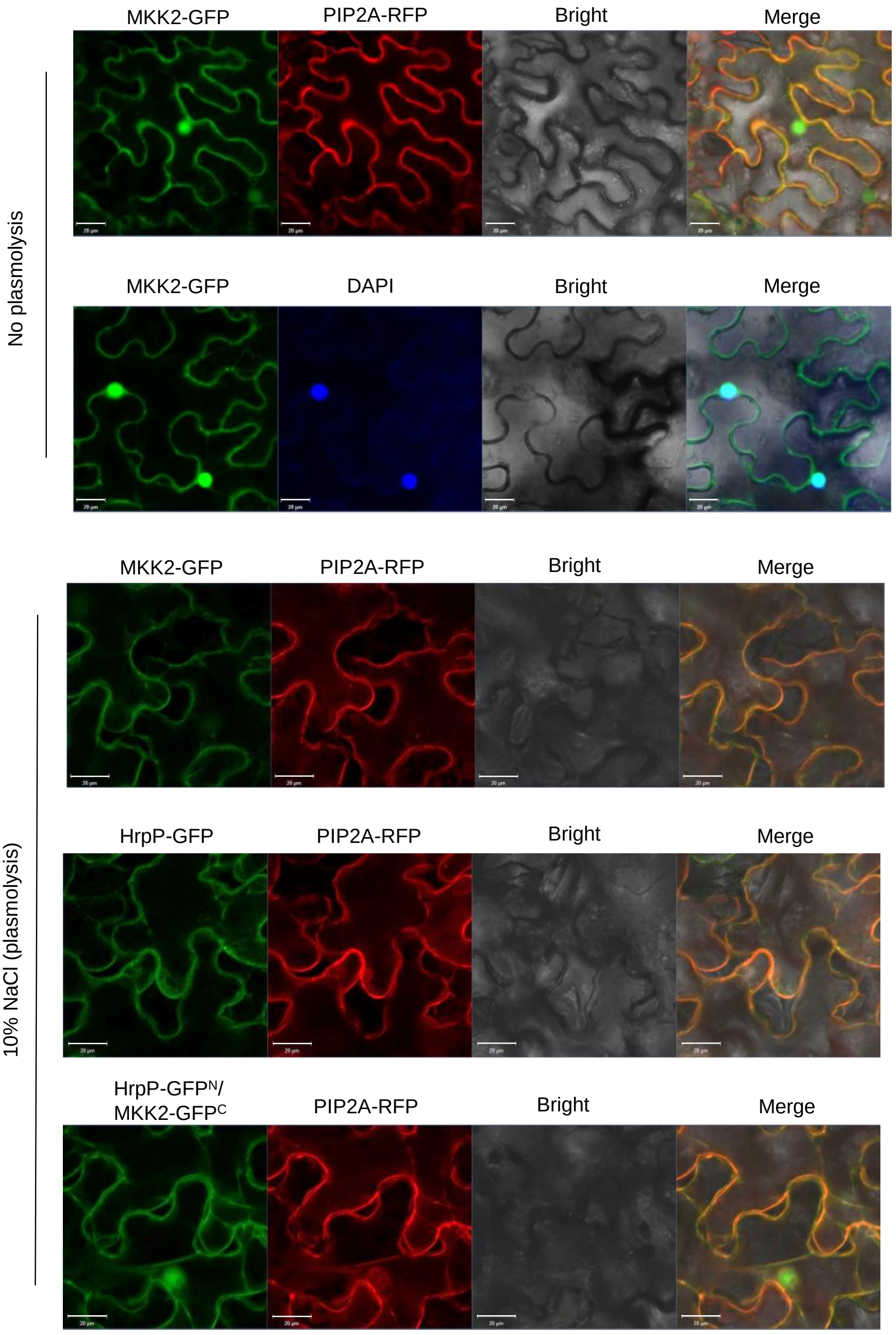

FIG S6 Subcellular localization of MKK2 and HrpP in plasmolyzed *N. benthamiana*.

For subcellular localization assay, MKK2-GFP fusion protein or HrpP-GFP fusion protein was expressed in *N. benthamiana* leaves by *Agrobacterium* infiltration. For BiFC assay, HrpP-GFP^{N} and MKK2-GFP^{C} were coexpressed in *N. benthamiana* leaves. PIP2A-RFP was used as the PM marker, and DAPI was used to stain nuclei. Fluorescence was detected by confocal microscopy at 2 dpi. 10% NaCl was infiltrated into leaves for plasmolysis assay. Scale bar = 20 μm. No plasmolyzed (30×). Plasmolyzed (40×).

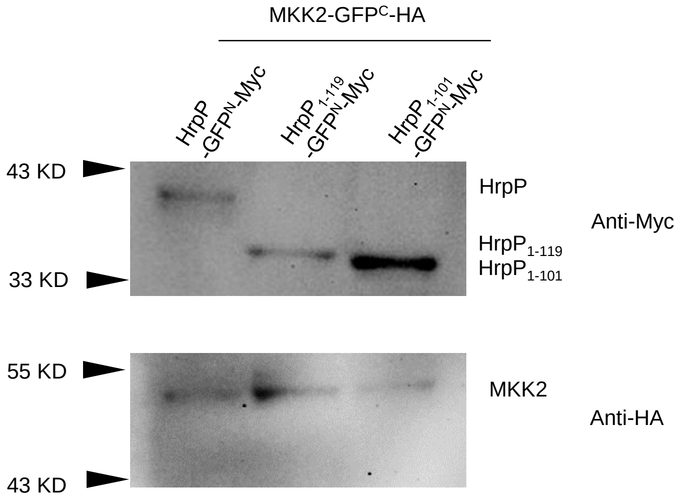

FIG S7 Western blot analysis of the target protein expression in BiFC .

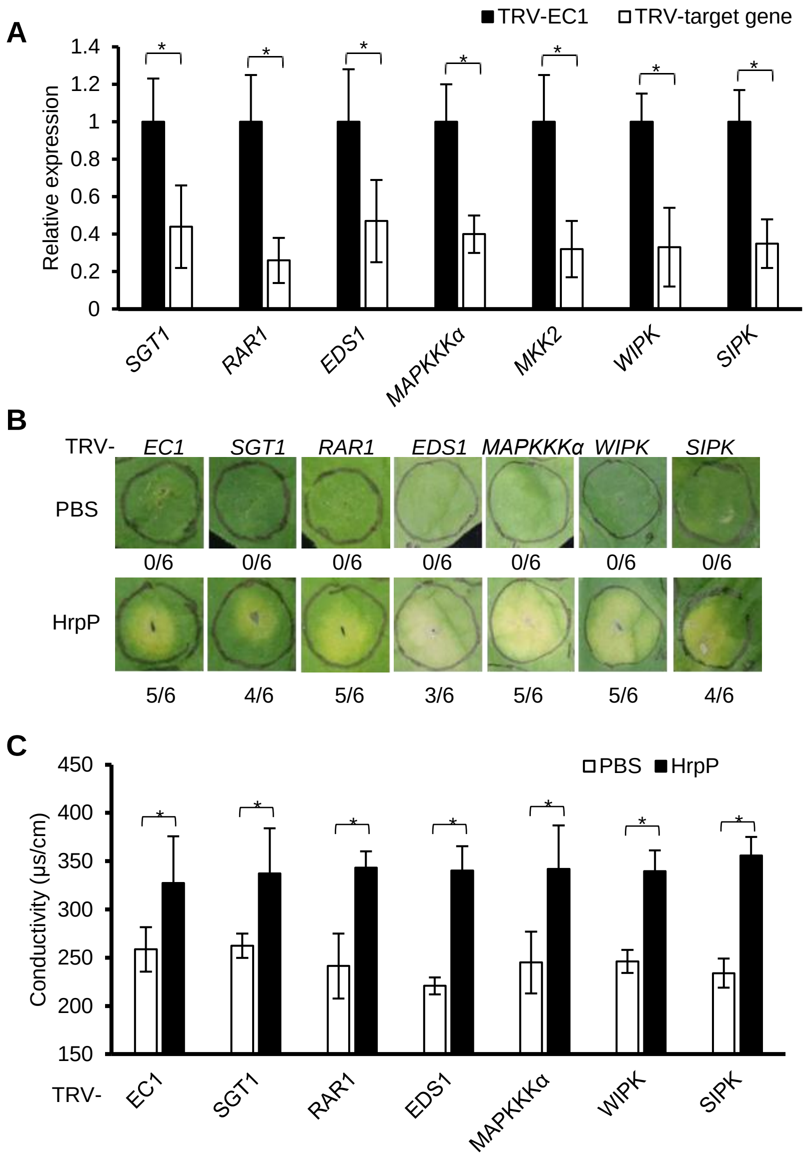

FIG S8 HrpP induces cell death independently of known cell death regulators.

(A) All selected genes were successfully silenced in *N. benthamiana*. Data were generated using *NbEF1α* as reference gene.

(B, C) PBS or 20 uM HrpP protein were infiltrated into the indicated *N. benthamiana* plants. Cell death was assessed by tissue phenotype at 2 dpi (B), and conductivity assay at 1 dpi (C).

Data shown are means ± standard deviation. * indicates the statistically significant difference (*t*-test) at *P*<0.05. All experiments were repeated at least three times and similar results were observed.
